# Differential Expression of Complement Markers in Normal and AMD Transmitochondrial Cybrids

**DOI:** 10.1371/journal.pone.0159828

**Published:** 2016-08-03

**Authors:** Sonali Nashine, Marilyn Chwa, Mina Kazemian, Kunal Thaker, Stephanie Lu, Anthony Nesburn, Baruch D. Kuppermann, M. Cristina Kenney

**Affiliations:** 1 Gavin Herbert Eye Institute, University of California Irvine, Irvine, California, United States of America; 2 College of Osteopathic Medicine, Touro University Nevada, Nevada, United States of America; 3 VA Medical Center Long Beach Hospital, Long Beach, California, United States of America; 4 Cedars-Sinai Medical Center, Los Angeles, California, United States of America; 5 Department of Pathology and Laboratory Medicine, University of California Irvine, Irvine, California, United States of America; University of Florida, UNITED STATES

## Abstract

**Purpose:**

Variations in mitochondrial DNA (mtDNA) and abnormalities in the complement pathways have been implicated in the pathogenesis of age-related macular degeneration (AMD). This study was designed to determine the effects of mtDNA from AMD subjects on the complement pathway.

**Methods:**

Transmitochondrial cybrids were prepared by fusing platelets from AMD and age-matched Normal subjects with Rho*0* (lacking mtDNA) human ARPE-19 cells. Quantitative PCR and Western blotting were performed to examine gene and protein expression profiles, respectively, of complement markers in these cybrids. Bioenergetic profiles of Normal and AMD cybrids were examined using the Seahorse XF24 flux analyzer.

**Results:**

Significant decreases in the gene and protein expression of complement inhibitors, along with significantly higher levels of complement activators, were found in AMD cybrids compared to Older-Normal cybrids. Seahorse flux data demonstrated that the bioenergetic profiles for Older-Normal and Older-AMD cybrid samples were similar to each other but were lower compared to Young-Normal cybrid samples.

**Conclusion:**

In summary, since all cybrids had identical nuclei and differed only in mtDNA content, the observed changes in components of complement pathways can be attributed to mtDNA variations in the AMD subjects, suggesting that mitochondrial genome and retrograde signaling play critical roles in this disease. Furthermore, the similar bioenergetic profiles of AMD and Older-Normal cybrids indicate that the signaling between mitochondria and nuclei are probably not via a respiratory pathway.

## Introduction

Age-related macular degeneration (AMD) is a blinding eye disease and one of the leading causes of vision loss in developed countries. Although several genetic and environmental factors contribute to AMD, major risk factors for AMD include cigarette smoking, family history of AMD, nutritional factors, hypertension, and cardiovascular diseases. The early form of AMD is characterized by formation of drusen deposits beneath the retina and can progress to the late form of dry AMD, which has extensive loss of the retina pigment epithelium (RPE), along with overlying retina, and results in loss of central vision. Approximately 10–15% of the AMD cases have wet macular degeneration, which is characterized by choroidal neovascularization, and accounts for approximately 90% of the severe vision loss caused by AMD. Although several drug treatments are in use for wet AMD, there is no proven medical treatment for dry AMD [1,2,3].

Mitochondrial dysfunction has been shown to be associated with the development and progression of AMD. Transmission electron microscopy has shown that mitochondria in the RPE cells have disrupted cristae and ruptured membranes [4]. Other studies have demonstrated that the mitochondrial (mt) DNA from AMD retinas are fragmented and damaged [5,6], which undoubtedly decreases the mitochondrial function. The human mtDNA is a double-stranded, circular molecule that encodes 37 genes and 13 proteins, which are critical for the electron transport chain and oxidative phosphorylation (OXPHOS) [7,8]. Most recently it has been reported that the mtDNA can also encode for short, biologically active peptides, called mitochondrial derived peptides (MDPs), which have anti-apoptotic and cyto-protective properties for neuronal cells and retinal ganglion cells [9,10,11]. Epidemiological studies have used mtDNA to study geographic origins of population by classifying individuals into haplogroups, based on the accumulation of specific single nucleotide polymorphisms (SNPs). Some mtDNA haplogroups, such as the H haplogroup, are protective against AMD, whereas the J, U, and T haplogroups are high risk for developing the disease [12]. By using the transmitochondrial cybrid model, where the nuclei are identical but the mtDNA varies, it has been shown that unique mtDNA variants within the different haplogroups can influence the expression of genes in the complement, inflammation and apoptosis pathways, which are major pathways in the pathogenesis of AMD [13].

Since 2005, it has been recognized that complement abnormalities play an important role in AMD. Genetic associations of numerous complement genes and defective regulation of the complement pathway increases an individual’s risk of developing advanced stages AMD [14]. Furthermore, recent studies have revealed that complement regulatory proteins, including complement factor H (CFH), C3, C5, C6, C7, C8, and C9, are molecular constituents of drusen, the hallmark deposits of extracellular material found between Bruch’s membrane and the retinal pigment epithelium, in AMD retinas. This suggests that there is local, complement-mediated inflammation in the diseased retina [15,16]. The complement system is a signaling pathway of the innate immune system that has been implicated in the pathology of several diseases with an immune component, such as multiple sclerosis, arthritis, Barraquer-Simons Syndrome, asthma, glomerulonephritis, and autoimmune heart disease. It is also known to play a role in neurodegenerative diseases such as Alzheimer's disease [17].

**Fig 1:**
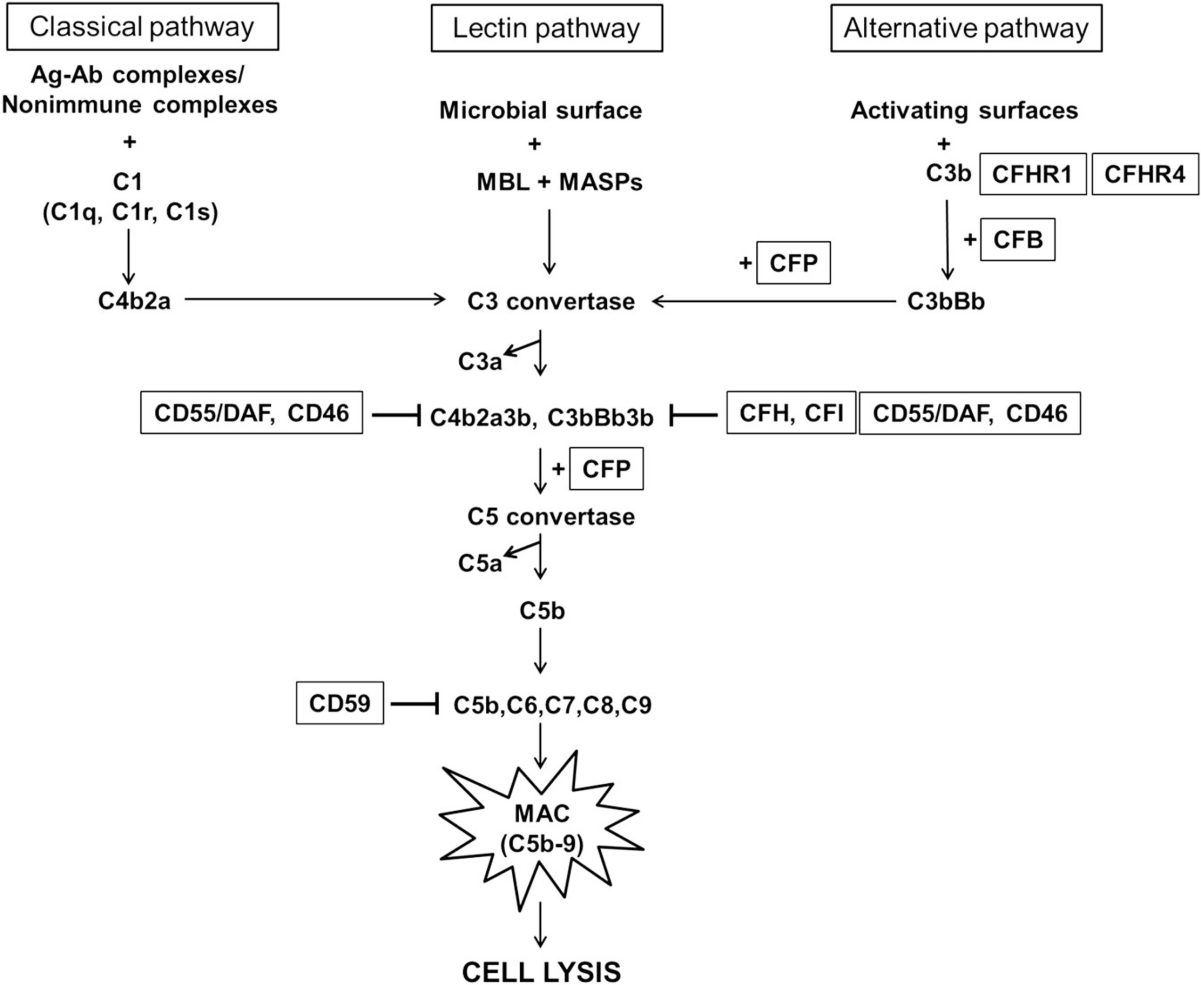
Overview of the complement cascade. The classical pathway is triggered by interaction of C1 with immune or non-immune complexes leading to conformational changes in C1q, activation of C1r and C1s, and subsequent assembly of C3 convertase. The lectin pathway is activated by binding of mannose-binding lectin (MBL) to mannose residues on the pathogen surface, which activates the MBL-associated serine proteases (MASPs), followed by formation of C3 convertase. Spontaneous hydrolysis of C3 initiates the alternative complement pathway. All three pathways of the complement cascade converge on the classical C3 convertase, which cleaves and activates component C3, forming C3a and C3b. This triggers a series of further cleavage and activation events, leading to cleavage of C5 into C5a and C5b, and eventual formation of the membrane attack complex (MAC), consisting of C5b, C6, C7, C8, and C9. MAC is the terminal cytolytic complex of the complement pathway; it causes osmotic lysis of target cells by forming transmembrane channels that disrupt the phospholipid bilayer of target cells. The complement system is tightly regulated by the following complement control proteins: CFH, CFHR1, CFHR4, CFB, CD55, CD59, CD46, CFI, and CFP.

Although many studies have demonstrated the involvement of complement factors in the pathogenesis of AMD, it has not been clear if the mitochondria from AMD subjects might have a modulating effect for expression of critical complement associated genes. Therefore, in our present study, we fused the AMD and age-matched normal platelets with Rho*0* ARPE-19 cells (lacking mtDNA) to create transmitochondrial cybrids to determine if mtDNA has any effect on the expression of the nuclear-encoded complement genes. All cybrid cell lines in this study had identical nuclei but contained mtDNA from either AMD subjects or age-matched normal subjects. Therefore, any changes in the gene or protein expression of complement markers (Fig 1) could be attributed to variants or changes within the mtDNA. Our results showed decreased RNA and protein levels of complement inhibitors and elevated expression of activators of the complement cascade. Our findings suggest that retrograde signaling from the AMD mitochondria plays a role in the activation of the complement pathway, such as that seen in AMD subjects. Furthermore, in our cybrid model, we used mitochondria that originated from the subject’s platelets, suggesting that the complement abnormalities observed in AMD patients may be systemic, and may not be just localized to the retina. Understanding the retrograde signaling (between the AMD mitochondria and nuclei) that regulates the complement cascade could help identify future therapeutic targets for the development of a treatment regime for AMD and perhaps allow for biological marker(s) for diagnosis.

## Material and Methods

### Ethics Statement

Research involving human participants was approved by the Institutional review board of the University of California, Irvine (#2003–3131). Written informed consent was obtained from all patients and clinical investigations were conducted according to the Declaration of Helsinki.

### Transmitochondrial Cybrids

Transmitochondrial cybrids were created using methods previously described [18]. The mitochondria-free APRE-19 (Rho*0*) cells were fused with platelets isolated from either AMD patients (n = 8) or age-matched normal individuals (n = 11, Table 1). 10 ml of peripheral blood was collected via venipuncture in tubes containing 10 mM EDTA. Blood platelets were isolated by a series of centrifugation steps, in tubes containing 3.2% sodium citrate, and final pellets were suspended in Tris buffered saline. Rho*0* cells were created by serial passages in low doses (50 ng/mL) of ethidium bromide. Cybrids were produced by polyethylene glycol fusion of platelets with Rho*0* ARPE-19 cells. Successful fusion of the mitochondria-rich platelets and Rho*0* ARPE-19 cells was verified by using polymerase chain reaction (PCR) with primers for MT-ND2.

**Table 1:** Epidemiology Information for AMD and Normal Cybrids Used in This Study.

Number	Cybrid #	Age	Gender	Sample type	Haplogroup
1	14–132	78	M	Older-Normal	H
2	14–135	75	F	Older-Normal	H
3	13–52	58	F	Older-Normal	H
4	15–152	69	M	Older-Normal	H
5	15–156	69	F	Older-Normal	U
6	14–131	79	M	Older-Normal	V
7	14–140	81	M	Older-Normal	V
8	14–137	77	M	Older-Normal	K
9	14–144	86	F	AMD	H
10	14–145	84	F	AMD	H
11	14–146	75	F	AMD	H
12	14–141	82	F	AMD	H
13	14–143	84	M	AMD	H
14	15–151	90	M	AMD	H
15	14–139	81	F	AMD	H
16	13–128	86	M	AMD	H
17	13–129	89	M	AMD	K
18	14–130	74	F	AMD	N
19	14–138	69	M	AMD	U
20*	10–04	33	M	Young-Normal	H
21*	10–07	49	M	Young-Normal	H
22*	11–10	30	M	Young-Normal	H
23*	11–35	30	F	Young-Normal	H

* Younger-Normal cybrid cultures were used only for the Seahorse flux analyses experiments

Tissue culture methods used for the Seahorse flux analyzer experiments were as follows: Cybrids were established from Younger-Normal individuals (n = 4, age 35.5 ± 4.6), Older-Normal individual (n = 6, age 71.7 ± 1.6) and Older-AMD subjects (n = 8, age 77.4 ± 2.7). The ages of the Young-Normal subjects were significantly different than the Older-Normal or Older-AMD (P < 0.0001) but there was no difference between the Older-Normal and Older-AMD ages (P = 0.12). The cybrid cells were plated at 50,000 cells/well and cultured overnight at 37°C under 5% CO_2_. Plates were then washed and placed 1 hr in a 37°C incubator under air in 500 μL of unbuffered DMEM (Dulbecco’s modified Eagle’s medium, pH 7.4), supplemented with 17.5 mM Glucose (Sigma, St Louis, MO), 200 mM L-glutamine (Invitrogen-Molecular Probes, Carlsbad, CA) and 10 mM sodium pyruvate (Invitrogen-Molecular Probes). These transmitochondrial cybrids were then grown in DMEM/Ham’s F12 1:1 (Invitrogen-Gibco, Grand Island, NY) cell culture medium containing 24 mM sodium bicarbonate, 10% dialyzed fetal bovine serum, and 1.0 mM sodium pyruvate. Passage 5 cybrids (n = 4–8) were used for all experiments.

Mitochondrial haplogroups of each cybrid were confirmed using PCR, restriction enzyme digestion and/or sequencing of mtDNA as described previously [19]. The SNPs defining the H haplogroup were T7028C and G73A. The U defining SNPs were positive for A12308G but negative for G9055A. The K haplogroup was positive for both A12308G and G9055A SNPs, V was positive for G4580A, and N was positive for T10873T [20].

### RNA Extraction and cDNA Synthesis

Normal and AMD cybrid cells were grown in 6-well plates at a density of 500,000 cells/well. Cells were trypsinized and pelleted after 24 hr, and RNA was extracted using the RNeasy Mini Kit (Qiagen, Valencia, CA, USA) as per the manufacturer’s protocol. The extracted RNA was quantified using NanoDrop 1000 Spectrophotometer (Thermoscientific, Waltham, Massachusetts, USA). 100 ng/uL of RNA was reverse transcribed into cDNA using Superscript VILO Master Mix (Catalog no. 11755–050, Invitrogen, Grand Island, NY). Diluted cDNA was stored at –20°C until use.

### Quantitative Real-time PCR

Quantitative real-time PCR (qRT-PCR) was performed using StepOnePlus Real-Time PCR system (Applied Biosystems, Carlsbad, CA) to study the expression of complement genes (CFH, CFHR4, CD55/DAF, CD59, CD46, CFI, and CFP, Table 2). QuantiTect Primer Assays (Qiagen) (Table 3) and Power SYBR® Green PCR Master Mix (Catalog no. 4367659, Life Technologies, Grand Island, NY) were used and each sample was run in triplicate. Specific housekeeper genes used are listed in Table 3. qRT-PCR data were analyzed using ΔΔCt method. ΔCt was the difference between the Cts (threshold cycles) of the target gene and Cts of the housekeeper gene (the reference gene). ΔCts of normal and AMD groups were compared for statistical analysis using the Student’s t-test in the GraphPad Prism 5.0 software program. ΔΔCt was calculated by subtracting ΔCt of the AMD group from ΔCt of the normal group. Fold change was calculated using the following formula: Fold change = 2^ΔΔCt^.

**Table 2:** Complement Markers’ Information.

Sr. No.	COMPLEMENT MARKER SYMBOL	COMPLEMENT MARKER NAME	FUNCTION
1	**CFH**	Complement Factor H	CFH has an inhibitory action and protects healthy cells by preventing activation of the complement system when it is not needed.
2	**CD55/DAF**	Complement decay accelerating factor	CD55 accelerates decay of target proteins, thereby disrupting the cascade and preventing damage to host cells.
3	**CD59**	Complement defense 59	CD59 is a potent inhibitor of the complement membrane attack complex (MAC) action.
4	**CD46/MCP**	Cluster of differentiation 46/ Membrane Cofactor Protein	CD46 acts as a cofactor for complement factor I, and regulates the complement pathway.
5	**CFI**	Complement Factor I	CFI cleaves and inactivates the complement components C4b and C3b, and it prevents the assembly of the C3 and C5 convertase enzymes.
6	**CFP**	Properdin/ Complement Factor P	CFP binds to many microbial surfaces and apoptotic cells and stabilizes the C3- and C5-convertase enzyme complexes in a feedback loop that ultimately leads to formation of the membrane attack complex and lysis of the target cell.
7	**CFB**	Complement Factor B	CFB is a component of the alternative pathway of complement activation. It helps in the assembly of C3 convertase.
8	**CFHR1**	Complement Factor H-related protein 1	CFHR1 competes with CFH and reduces complement inhibition, thereby acting as a complement activator molecule.
9	**CFHR4**	Complement Factor H-related protein 4	CFHR4 helps in the activation of the complement cascade by a) enhancing opsonization and b) helping in the assembly of C3 convertase

**Table 3:** Complement Genes’ Information.

Sr. No.	GENE	Gene Bank Accession no.	QIAGEN Primer Name	QIAGEN Primer Catalog #	Housekeeper gene used
1	**CFH**	NM_000186	Hs_CFH_1_SG QuantiTect Primer Assay	QT00001624	HMBS
2	**CFHR4**	NM_001201550, NM_001201551, NM_006684	Hs_CFHR4_1_SG QuantiTect Primer Assay	QT00495292	ALAS variant(va)1
3	**CD55/DAF**	NM_000574, NM_001114543, NM_001114544, NM_001114752, NM_001300902, NM_001300903, NM_001300904	Hs_DAF_1_SG QuantiTect Primer Assay	QT00099190	HPRT1
4	**CD59**	NM_000611, NM_001127223, NM_001127225, NM_001127226, NM_001127227, NM_203329, NM_203330, NM_203331	Hs_CD59_1_SG QuantiTect Primer Assay	QT00035952	TUBB
5	**CD46/MCP**	NM_002389, NM_153826, NM_172350, NM_172351, NM_172352, NM_172353, NM_172358, NM_172359	Hs_MCP_1_SG QuantiTect Primer Assay	QT00073689	HPRT1
6	**CFI**	NM_000204	Hs_CFI_1_SG QuantiTect Primer Assay	QT00213794	ALAS va1
7	**CFP**	NM_001145252, NM_002621	Hs_CFP_1_SG QuantiTect Primer Assay	QT00010514	ALAS va1

### Protein Extraction

For protein extraction, normal and AMD cybrid cells were plated in 6-well plates at a density of 500,000 cells /well and were allowed to grow for 72 hr. Then, cell culture medium was removed and RIPA cell lysis buffer (Catalog no. 89900, Life Technologies) containing protease inhibitor cocktail was added to each well of the 6-well plate, and cells were scraped and transferred to microfuge tubes. Cell lysate was then centrifuged for 15 minutes at 13,000 rpm. Supernatant was transferred to a new microfuge tube and was mixed well and 10 μL of supernatant aliquoted to another tube for protein measurement. The concentration of proteins was measured using Bio-Rad Dc protein assay system (Bio-Rad Laboratories, Richmond, CA) according to the manufacturer's instructions.

### Western Blotting

A total of 10–15 μg protein per sample was loaded in the wells of 4–12% Bolt mini gels (Life Technologies) and was separated using SDS-PAGE electrophoresis for 1 hr at 100V. The gels were then transferred onto PVDF membranes. Following transfer, the membranes were blocked in 5% milk for 1 hr. The blots were incubated overnight at 4°C with primary antibodies (Table 4). Blots were then washed with 1x TBST and incubated with the respective secondary antibodies for 1 hr at room temperature. All primary and secondary antibodies were diluted in 1x TBST, except the CFH primary antibody and the Donkey pAb to Goat IgG (HRP), which were diluted in 5% milk. After secondary antibody incubation, the blots were washed with 1x TBST. Protein bands were detected using Clarity™ Western ECL Blotting Substrate (Catalog no.1705060, Bio-Rad). β-actin antibody was used as a housekeeper protein normal. Protein bands were visualized using the Versadoc imager, and quantified using Image J software.

**Table 4:** Complement Proteins’ Western Blotting Information.

Sr. No.	Protein	Predicted Protein band size	Primary Antibody used	Primary antibody dilution used	Primary Antibody Species reactivity	Secondary antibody used	Housekeeping protein used
1	**CFH**	**139 kDa**	Goat Anti-Factor H Polyclonal antibody ab36134 (Abcam)	1:4000	Human	Dnk pAb to Goat IgG (HRP) ab97120 (ABCAM)	beta-actin antibody GTX 110564 (Genetex)
2	**CD55**	**41 kDa**	Rabbit Polyclonal antibody to CD55 GTX113170 [N1C2] (Genetex)	1:1000	Human	Rabbit IgG Ab (HRP) GTX 213110–01 (Genetex)	beta-actin antibody GTX 110564 (Genetex)
3	**CD59**	**14 kDa (Monomer) ~ 40kDa (Glysolysated form)**	Rabbit Anti-Ly6c/CD59 Polyclonal antibody bs-12327R (Bioss USA) OWL ID# 37854 (One world Lab)	1:1000	Human	Rabbit IgG Ab (HRP) GTX 213110–01 (Genetex)	beta-actin antibody GTX 110564 (Genetex)
4	**CFI**	**65 kDa**	Rabbit Anti-Factor I heavy chain Polyclonal antibody bs-10339R (Bioss USA) OWL ID# 37844 (One world Lab)	1:1000	Human, Mouse, Rat	Rabbit IgG Ab (HRP) GTX 213110–01 (Genetex)	beta-actin antibody GTX 110564 (Genetex)
5	**CFP**	**51 kDa**	Rabbit Anti-Properdin Polyclonal antibody bs-8306R (Bioss USA) OWL ID# 37824 (One world Lab)	1:1000	Human, Mouse, Rat	Rabbit IgG Ab (HRP) GTX 213110–01 (Genetex)	beta-actin antibody GTX 110564 (Genetex)
6	**CFB**	**85 kDa**	Goat Anti-factor B Polyclonal antibody # AF2739 (RD Systems)	1:1000	Human	Dnk pAb to Goat IgG (HRP) ab97120 (ABCAM)	beta-actin antibody GTX 110564 (Genetex)
7	**CD46**	**44 kDa**	Goat Anti-CD46 Polyclonal antibody # AF2005 (RD Systems)	1:1000	Human	Dnk pAb to Goat IgG (HRP) ab97120 (ABCAM)	beta-actin antibody GTX 110564 (Genetex)
8	**CFHR4**	**65 kDa**	Mouse anti-Factor H-related 4 (CFHR4) Monoclonal antibody #MAB5980 (RD Systems)	1:1000	Human	Anti-mouse IgG, HRP-linked antibody #7076 (CST)	beta-actin antibody GTX 110564 (Genetex)
9	**CFHR1**	**37 kDa**	Mouse anti-Factor H-related 1 (CFHR1) Monoclonal antibody #MAB4247 (RD Systems)	1:1000	Human	Anti-mouse IgG, HRP-linked antibody #7076 (CST)	beta-actin antibody GTX 110564 (Genetex)

### Bioenergetic Profiles for the Normal and AMD Cybrids

The real-time bioenergetic profiles for the Older-AMD, Older-Normal and Younger-Normal cybrids were measured with the Seahorse XF24 flux analyzer (Seahorse Bioscience–Agilent Technologies, Billerica, MA) as described previously [21]. There was sequential injection into the wells of Oligomycin (1 μM final concentration, which blocks ATP synthase to assess respiration required for ATP turnover), FCCP (1 μM final concentration, a proton ionophore which induces chemical uncoupling and maximal respiration), and Rotenone plus Antimycin A (1 μM final concentration of each, completely inhibits electron transport to measure non-mitochondrial respiration). Data from each well were normalized by measuring total protein. Total protein was isolated with RIPA lysis buffer (Millipore, Billerica, MA) containing protease inhibitor (Sigma, St. Louis, MO) and phosphatase arrest (G-Biosciences, St. Louis, MO). Isolated protein was mixed with Qubit buffer and measured with Qubit 2.0 fluorometer (Invitrogen, Grand Island, NY). The analyses of the bioenergetic measurements provide values for the oxygen consumption rates (OCR), ATP turnover, spare respiratory capacity, proton leak and non-mitochondrial respiration, which were determined from the OCR traces. In addition, the extracellular acidification rates (ECAR, representing glycolysis) were also measured.

The Older-AMD (n = 8), Older-Normal (n = 6) and Younger-Normal (n = 4) samples, each representing different individuals, were run in biological triplicates and the entire experiment was repeated twice. The data for each assay were analyzed using the Seahorse Data Analysis Software Program (Seahorse Bioscience–Agilent Technologies). The final analyses were done by exporting all data to GraphPad Prism 5 (GraphPad Software, La Jolla, CA, USA), then normalizing the values and analyzing the Older-AMD versus Older-Normal versus Younger-Normal cybrids. Statistical significance was determined by performing two-tailed Student’s t-tests and P≤ 0.05 was considered significant in all experiments. All values are presented as Mean ± SEM.

### Statistical analysis

GraphPad Prism 5.0 software was used for statistical analysis. Results between normal and AMD groups were analyzed for differences using the Student’s t-test. Differences with P values ≤ 0.05 were considered to be statistically significant.

## Results

### Complement Gene Expression

We performed qRT-PCR for seven genes related to the complement pathway for the AMD cybrids and age-matched normal cybrids and found significant differences between the groups. We observed a significant decrease in the expression of complement inhibitors, i.e., CFH (Fold change = 0.71, P = 0.02), CD55 (Fold change = 0.50, P = 0.03), CD59 (Fold change = 0.66, P = 0.02), CD46 (Fold change = 0.72, P = 0.04), and CFI (Fold change = 0.55, P = 0.03), in AMD cybrids compared to Older-Normal cybrids (Table 5). In contrast, the expression of CFP, a positive regulator of the complement pathway, was significantly increased (Fold change = 2.18, P = 0.03) in AMD cybrids compared to the normal cybrids. However, there was an increase (Fold change = 24.16, P = 0.01) in the expression of CFHR4 gene, a variant of CFH, in the AMD group. These data demonstrate that the cells which contain mtDNA from AMD subjects have lower RNA levels for inhibitors of the complement pathway and higher expression for activators of the alternative complement pathway.

**Table 5:** Complement Gene Expression Data.

Sr. No.	Gene	Sample size (n)	P-value	Fold Change	Delta delta Ct
1	**CFH**	n = 5–6	0.02	0.71	-0.49 ± 0.20
2	**CFHR4**	n = 6	0.01	24.61	4.62 ± 1.76
3	**CD55**	n = 6	0.03	0.50	-0.99 ± 0.43
4	**CD59**	n = 6–8	0.02	0.66	-0.61 ± 0.25
5	**CD46/MCP**	n = 6–8	0.04	0.72	-0.48 ± 0.22
6	**CFI**	n = 6	0.03	0.55	-0.85 ± 0.39
7	**CFP**	n = 6	0.03	2.18	1.12 ± 0.50

### Expression of Complement Proteins

**Fig 2:**
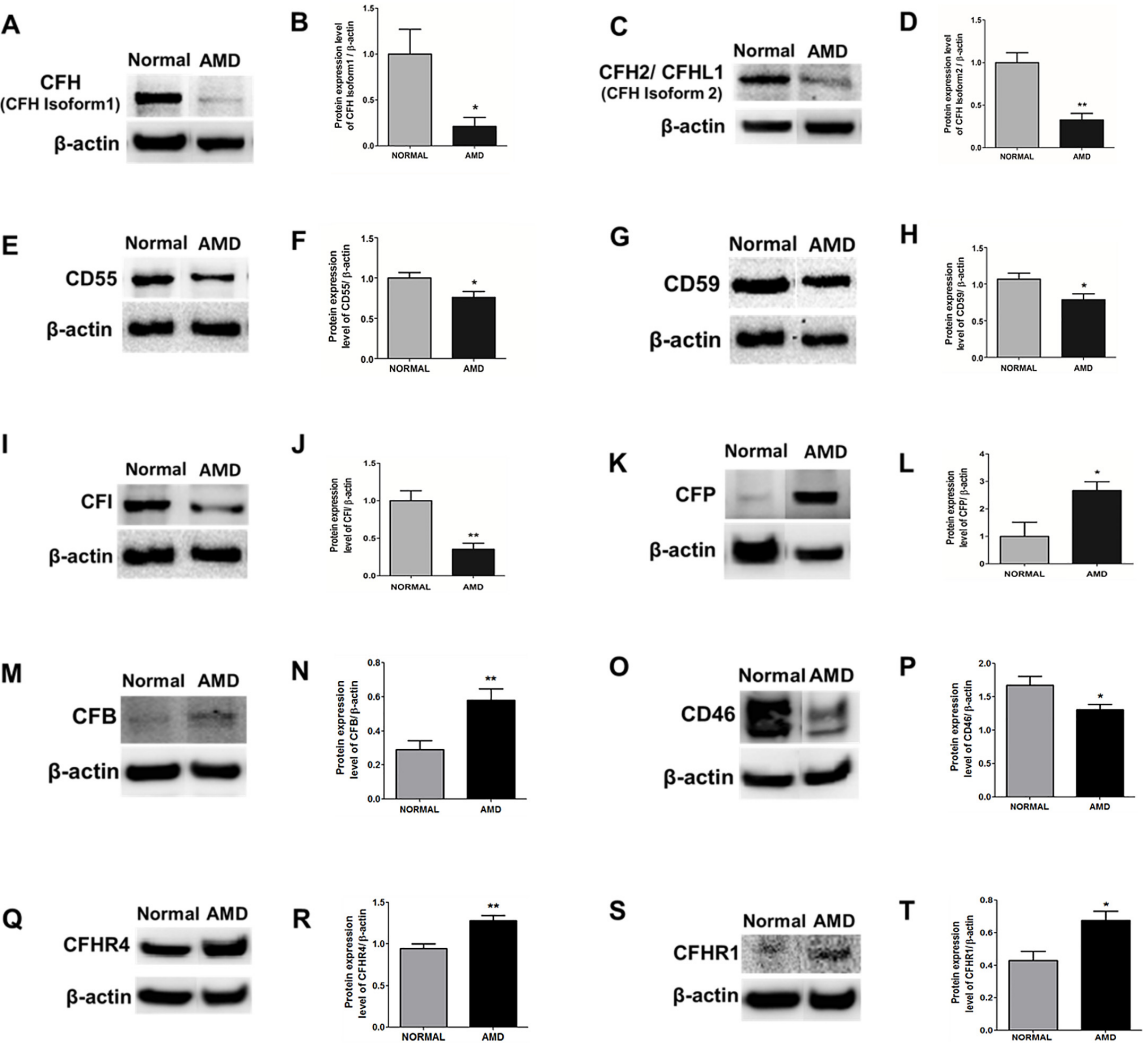
Western blot analysis of complement proteins. Decreased protein levels of CFH, CFHL1, CD55, CD59, CFI, and CD46, and increased levels of CFP, CFB, CFHR4, and CFHR1 protein in AMD cybrids. (A, C, E, G, I, K, M, O, Q, S) Representative Western blots of CFH, CFHL1, CD55, CD59, CFI, CFP, CFB, CD46, CFHR4, and CFHR1 respectively. (B, D, F, H, J, L, N, P, R, T) Graphs showing quantitation of CFH, CFHL1, CD55, CD59, CFI, CFP, CFB, CD46, CFHR4, and CFHR1 proteins in Older-Normal and AMD cybrids. * P < 0.05, ** P < 0.01. n = 4–5. Data were analyzed using Student’s T-test.

The protein expression levels of complement markers, CFH, CFI, CD55, CD59, CD46, CFB, CFP, CFHR4, and CFHR1 were determined using Western blot analysis. Consistent with the qRT-PCR results, protein expression levels for complement proteins were significantly different between Older-Normal and AMD cybrids (Fig 2, Table 6). In the AMD cybrids, there were significant decreases in the protein levels for the CFH (Isoform 1; 21%, P = 0.03); CFHL1 (Isoform 2; 33%, P = 0.001); CD55 (76%, P = 0.04); CD59 (79%, P = 0.048); CD46 (78%, P = 0.04) and CFI (36%, P = 0.003), compared to Older-Normal cybrids that had been normalized to 100%. In contrast, the AMD cybrids showed a significant increase of CFP (267%, P = 0.03); CFB (199.8%, P = 0.007); CFHR4 (135%, P = 0.002) and CFHR1 (196%, P = 0.02) when compared to Older-Normal cybrids that had been normalized to 100%. These results validate our gene expression data and are consistent with activation of the complement cascade in AMD cybrids. Moreover, since all nuclei in the cybrids are identical, it suggests that the changes in the gene and protein expression of complement markers can be attributed to different retrograde signaling from the AMD versus the Older-Normal mtDNA variants.

**Table 6:** Complement Protein Expression Data.

Sr. No.	Protein	Observed protein band size	Sample size	P-value
1	**CFH**	~139 kDa and ~50 kDa	n = 4	0.03
2	**CD55**	41 kDa	n = 5	0.04
3	**CD59**	~ 40 kDa	n = 4	0.048
4	**CFI**	65 kDa	n = 5	0.003
5	**CFP**	51 kDa	n = 4	0.03
6	**CFB**	85 kDa	n = 6	0.007
7	**CD46**	44 kDa	n = 6	0.04
8	**CFHR4**	65 kDa	n = 7	0.002
9	**CFHR1**	37 kDa	n = 4–5	0.02

### Bioenergetic Profiles for the Normal and AMD Cybrids

**Fig 3:**
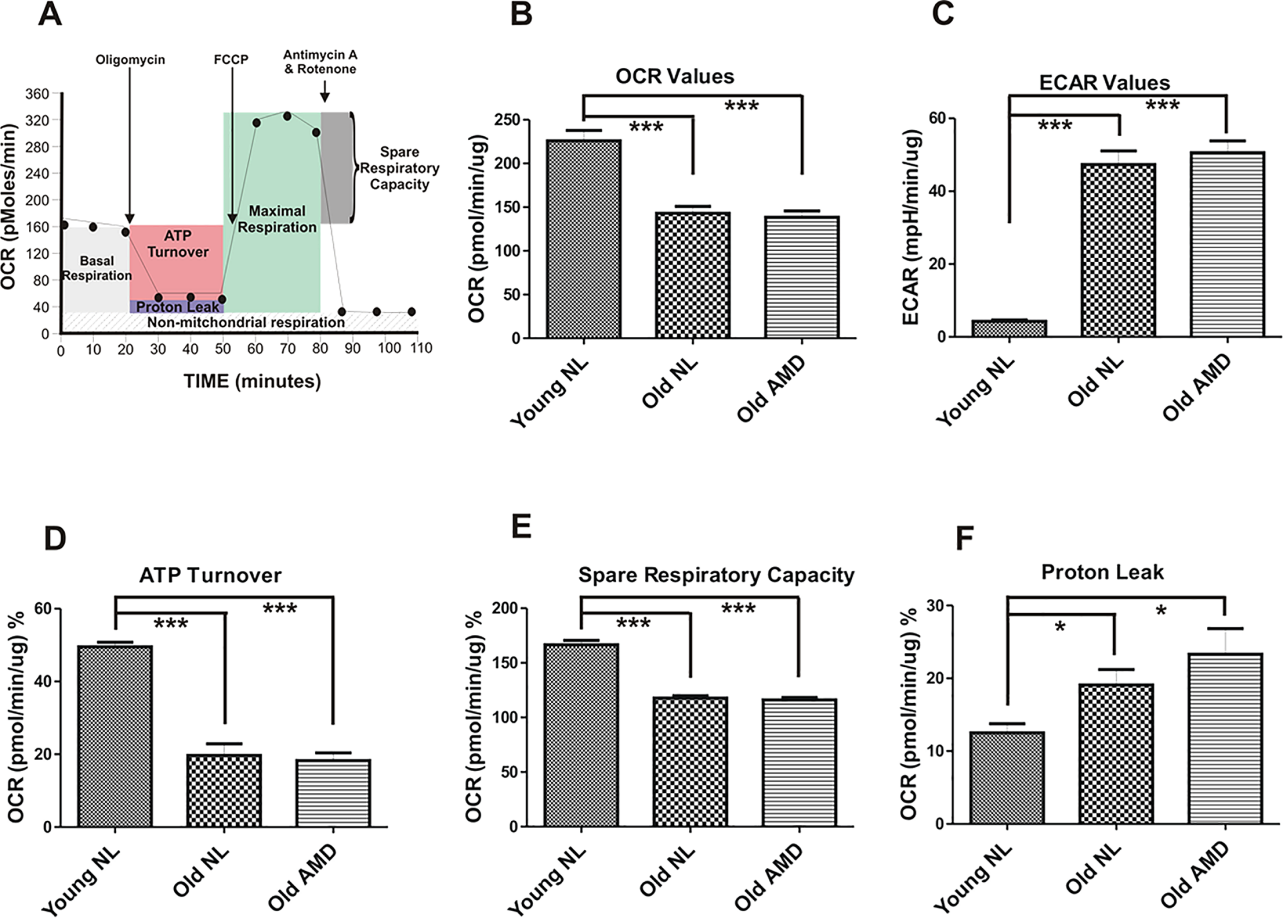
Bioenergetic profiles for Normal and AMD cybrids. (A) Representative bioenergetic profile measured by the Seahorse XF24 flux analyzer. This figure shows OCR and ECAR patterns after sequential treatment with oligomycin (1 μM), FCCP (1 μM) and Antimycin A plus Rotenone (1 μM), demonstrating the regions that define basal aerobic respiration, ATP turnover, maximal respiration, and spare respiratory capacity. (B), (C), (D), (E), (F) Graphs comparing the OCR, ECAR, ATP turnover, spare respiratory capacity, and proton leak values, respectively, in Young-Normal (NL), Old-Normal, and Old-AMD cybrids. These data are presented as a percent response from the basal readings, with non-mitochondrial respiration subtracted out and the basal respiratory rate normalized to 100%.

The Seahorse XF24 flux analyzer was used to compare the bioenergetic profiles for Young-Normal, Older-Normal and AMD cybrids (Fig 3) by measuring oxygen consumption rate (OCR), ATP turnover, maximal respiration, spare respiratory capacity, and proton leak in the cybrids (Fig 3A). The baseline OCR value for the Young-Normal cybrids was significantly higher (226.0 ± 11.75 pmol/min/ug) compared to the Older-Normal (143.1 ± 7.97 pmol/min/ug, P < 0.001) or the Older-AMD cybrids (138.5 ± 7.3 pmol/min/ug, P < 0.001, Fig 3B). However, the Older-Normal and Older AMD cybrids were not significantly different from each other (P = 0.67).

The ECAR represents the glycolysis occurring in the cultures. The Older-AMD cybrids (50.56 ± 3.2 mpH/min/ug, P < 0.0001) and Older-Normal cybrids (47.32 ± 3.7 mpH/min/ug, P < 0.0001) had higher levels of ECAR compared to the Young-Normal cybrids (4.27 ± 0.41 mpH/min/ug) but were not significantly different from each other (P = 0.52) (Fig 3C). Comparing the OCR: ECAR ratios showed significantly higher values for the Younger-Normal cybrids (8.14 ± 0.88 mpH/min/ug) compared to the Older-Normal (2.56 ± 0.28 mpH/min/ug, P < 0.001) or Older-AMD (2.34 ± 0.880.32 mpH/min/ug, P < 0.001), and again, there was no difference in the OCR: ECAR ratio between the Older-Normal and Older-AMD cybrids (P = 0.63).

The percentage ATP turnover rates was higher for the Young-Normal (49.5 ± 1.28 pmol/min/ug) compared to the Older-Normal cybrids (19.67 ± 3.18 pmol/min/ug, P < 0.0001) and the Older-AMD cybrids (18.27 ± 2.09 pmol/min/ug, P < 0.0001). There were no differences in ATP turnover between the Older-Normal and Older-AMD cybrids (P = 0.71) (Fig 3D). The Spare Respiratory Capacities were similar between in the Older-Normal (117.8 ± 2.09 pmol/min/ug) and Older-AMD cybrids (116 ± 2.29 pmol/min/ug, P = 0.57), while the Young-Normal cybrids was significantly higher (166.5 ± 4.09 pmol/min/ug, P < 0.0001) (Fig 3E). Finally, the Older-AMD cybrids (23.31 ± 3.5 pmol/min/ug, P = 0.01) and Older-Normal cybrids (19.08 ± 2.14 pmol/min/ug, P = 0.01) had higher levels of Proton Leak compared to the Young-Normal cybrids (12.5 ± 1.26 pmol/min/ug) but were not significantly different from each other (P = 0.33) (Fig 3F). These data suggest that the decline in the bioenergetic profile is due to the increased age of the subject and not related to presence of AMD in the person.

## Discussion

### Inhibitors of Complement Pathway

CFH was the first complement gene identified as high risk for AMD [22,23,24]. Individuals with the CFH rs10611770 variant (Tyr402His) have a 5-fold increased risk of AMD [25], and there are many minor CFH variants associated with AMD [26,27]. Other complement components (CFI, CFB, C3 and CFHR1/ CFHR3) are also high risk for AMD [28,29,30,31,32]. Therefore, understanding mechanisms for complement pathway regulation are critical for development of future therapies for AMD.

Our study showed that even though the AMD and age-matched Normal cybrids have identical nuclei, the gene and protein expression levels of complement inhibitors (CFH, CD55/DAF, CD59, CD46 and CFI) were decreased in AMD cybrids, while the activators (CFP, CFB, CFHR4, and CFHR1) were elevated compared to age-matched Normal cybrids. This is significant because complement activation plays a significant role in development of AMD [33,34,35,36]. The down-regulation of CFH transcription in AMD cybrids compared to age-matched Normal cybrids was supported by Western blot analyses demonstrating lower levels for the ~139 kDa CFH protein band (CFH isoform 1, representing the canonical sequence, p = 0.03) and also the ~50 kDa protein band (CFHL1 or CFH isoform 2, p = 0.001). The CFHL1 sequence differs from the canonical CFH sequence in two regions: a) 446–449: KTCS → SFTL, and b) 450–1231: Missing. Our findings are consistent with other studies showing that mtDNA can modulate the expression of genes associated with complement and inflammation pathways [37,38,39,40]. In our cybrid model, the wildtype ARPE-19 cells, and subsequent Rho*0* cells, are heterozygous for the CFH gene (one high risk C allele and one wildtype T allele), as determined by allelic discrimination (data not shown). Therefore, the AMD and Older-Normal cybrids possess the same CFH allelic pattern, so the differences in CFH levels are not due to variations in the nuclear genome but more likely are due to influencing factors of the AMD mitochondria.

In addition to CFH, the AMD cybrids showed lower levels of CFI and CD55/DAF compared to Older-Normal cybrids. C3b, an important opsonin of the complement cascade, is inactivated by CFH and its cofactor CFI is a regulator of C3b cleavage (Fig 1). Therefore, the lower levels of both CFH and CFI in the AMD cybrids suggest that the C3b component would be activated in those cells compared to the cybrids with mitochondria from normal subjects. CD55/DAF blocks complement activation by interacting with C3b and C4b fragments generated during activation of C3 (alternative complement pathway) and C4 (classical complement pathway), which in turn disrupts the amplification of convertases. Previous studies in mice have shown considerable protection of ocular tissues by adenovirus-delivered human CD55 [41]. Again, lower RNA/protein levels of CD55/DAF found within the AMD cybrids suggest a role for mitochondrial genome influencing, by yet unknown mechanisms, of the nuclear genes.

In AMD cybrids, the RNA and protein levels for CD59 were also significantly lower, which is important because of the CD59 inhibitory functions on the MAC complex. CD59 is a membrane-bound, regulatory protein that binds to the C5b-8 complex, thereby limiting the incorporation of multiple copies of C9 into MAC, and blocking lyses of target cells. The RPE cells of AMD retinas have elevated MAC deposits, which would be associated with higher levels of focal inflammation, a common feature of AMD [42].

Finally, the AMD cybrids also had lower transcription levels for CD46 or Membrane Cofactor Protein (MCP), an inhibitor that inactivates C3b and C4b. A recent study has shown that CD46 deficient mice are more susceptible to laser-induced choroidal neovascularization than wild-type mice [43]. It is significant that in the AMD cybrids, multiple inhibitors of the complement system are decreased. These inhibitors protect healthy cells from complement attack [44,45,46], so that having lower levels would increase the complement activating levels in the retinal cells and may be a contributing factor to the low-grade, chronic inflammation associated with AMD retinas.

### Activators of Complement Pathway

The AMD cybrids have increased levels of complement activators compared to age-matched normal cybrids. For example, levels of properdin (CFP), a key positive regulatory protein that stabilizes the C3bBb complex [47], and complement factor B (CFB), a complement activator molecule, were significantly higher in AMD cybrids. CFB can be localized to drusen [48] and upon activation of the alternative complement pathway, it is cleaved by complement factor D into two fragments: Ba, the noncatalytic subunit and Bb, the catalytic subunit. Bb, a serine protease, associates with C3b to form the C3 convertase [49].

The family of CFHR proteins is important in a number of diseases, including AMD, IgA nephropathy, and systemic lupus erythematosus [50,51,52]. In the present study, the gene expression and protein levels of CFH-related protein 4 (CFHR4) were increased significantly in AMD cybrids compared to age-matched normal cybrids. Studies have shown that CFHR4 has two different roles; it can act as a cofactor for factor H in C3b inactivation but it also can act as an activator. For example, Hebecker *et al*. demonstrated that CFHR4 is important in the activator role as it assists in the assembly of an active C3 convertase via the Activator Protein (AP) [53]. Furthermore, CFHR4 is a known ligand for C-reactive protein (CRP), binds to fluid-phase C3b and recruits CRP to necrotic cells, thereby enhancing their opsonization [54]. Therefore, CFHR4 activates complement via two mechanisms: a) binding to CRP and enhancing opsonization [55], and b) assembly of C3 convertase via the AP [56,57]. We speculate that in the AMD cybrids, the activator role for CFHR4 may be more important than its inhibitory functions and the higher CFHR4 levels may possibly represent a feedback loop to compensate for lower CFH levels. However, additional studies are needed to clarify the mechanisms by which the AMD mitochondria influence these activators.

Previous studies have shown that CFH-related protein 1 (CFHR1) is an isoform that confers risk for AMD [48]. Furthermore, individual homozygous deletions in CFHR1 and CFHR3 genes were protected against AMD development [48]. CFHR-1 is also involved in immune evasion of pathogens because it competes with the CFH for binding site on microbial surfaces [58,59]. In our study, the AMD cybrids demonstrated significantly higher levels of CFHR1 protein compared to normal cybrids. In the AMD cybrids, decline in complement inhibitors and increased levels of the complement activators suggests that in an AMD individual with these mitochondria may have higher systemic levels of complement activation. This is consistent with studies showing that in AMD subjects, there are significant elevations of serum C3a des Arg, a marker for complement activation, suggesting a systemic low grade inflammation in these patients [60]. Interestingly, the elevated serum levels of activated complement (as measured by ratio of C3 and its catabolic fragment C3d (C3d/C3)) were not correlated with the presence of known AMD high-risk complement genes [61,62]. Patients with advanced AMD also had elevated levels of Bb and C5a complement activation fragments that correlated to both smoking and increased basal metabolic index [63], again supporting the presence of systemic low grade inflammation in AMD subjects. Furthermore, evidence suggests that mitochondria play a role in this chronic inflammation because AMD subjects treated with oral zinc, a mediator for mitochondrial functions [64], showed a steep decrease of serum complement activation (C3d/C3 ratio). In future studies, it will be important to clarify the role of mtDNA in complement activation not only for AMD patients but also other diseases where complement plays a significant role in pathogenesis.

In the present study, we examined the gene and protein expression of complement markers only in Older-AMD and Older-Normal cybrid samples, and not in Young-Normal cybrids. However, our previous transmitochondrial cybrids studies using younger cybrids showed significantly decreased expression of CFH, CF55/DAF and CD59 in cybrids containing the mtDNA J haplogroup (high-risk for AMD) compared to the cybrids with H haplogroup (lower risk for AMD) [65]. For the AMD cybrid study, the numbers of high risk J haplogroup cybrids were too low for analyses, as the J haplogroup is found in only 6–8% of the European-origin population. In contrast, the H haplogroup is found in approximately 35–40% of the population so many more H cybrids have been generated. The present experiments were conducted using mainly H cybrids because we wanted to compare AMD and controls samples with similar haplogroups. The majority of Older-Normal cybrids were H or V (6/8, both are part of HV subclade), U haplogroup (1/8) or K (1/8) haplogroups. The majority of AMD cybrids were H (8/11), with U (1/11), K (1/11) and N (1/11) haplogroup cybrids also being studied. Collecting and generating high risk J cybrids is ongoing and will be the focus of another study once adequate numbers of cybrids are generated.

It should be noted that the present study was performed using subconfluent cells rather than fully differentiated monolayers cultured for 2–3 weeks. With the longer incubation periods, the RPE cells develop polarity, pigmentation, specific ion channels, fluid transport, well developed tight junctions and transepithelial resistance (TER) [66,67]. Also, in these cultures, complement activation leads to decreased TER, elevated matrix metalloproteinase activities and altered expression of angiogenesis-related genes [68]. The differentiated RPE cells isolated from aged donor eyes are more susceptible to oxidative stress than cells isolated from young donors [69]. Since the subconfluent and differentiated monolayer cultures can have different gene and proteins expression levels, future studies will include conducting similar experiments using the fully differentiated AMD and Older-Normal cybrids to confirm the differences.

### Bioenergetics of AMD and Normal Cybrids

Analyses using the Seahorse flux analyzer showed that the cybrids possessing mitochondria from Young-Normal individuals had significantly higher OCR values (indicating higher levels of OXPHOS, an efficient mechanism of ATP production) and ATP turnover, compared to both the Older-Normal and Older-AMD cybrids. In contrast, higher levels of ECAR were found in the Older-Normal and Older-AMD cybrids compared to Young-Normal cybrids, indicating that the old cybrids compensate for loss of mitochondrial function with increase in glycolysis.

Our findings are consistent with studies showing that mitochondrial efficiency declines with age and that glycolysis, as a mode of energy production, increases [70]. For example, Tombran-Tink and colleagues have demonstrated a correlation between aging and increased impairment of mitochondrial functions [71], showing that primary human RPE cells from older subjects make less ATP in vitro, and have decreased levels of mitochondrial membrane potential and ROS production. Likewise, using Seahorse extracellular flux assays to examine mitochondrial metabolism, Rohrer and coworkers demonstrated a complete loss of reserve capacity in aged primary human RPE cells [72]. Since the bioenergetic profiles of the Older-AMD cybrids and Older-Normal cybrids were similar to each other, it suggests that ATP levels or modes of energy production were not influencing the expression of complement components.

While mitochondrial-nuclear interactions have been recognized for many years [73,74,75,76], most of the focus has been on the influence of the nuclear genome upon mitochondrial functions, because the majority of proteins necessary for mitochondrial activities are actually encoded from nuclear genes. It has however, been accepted that mitochondrial damage/dysfunction can mediate changes in nuclear transcription. For example, in murine C2C12 myocytes, mitochondrial dysfunction leads to activation of calcineurin (Cn), NFAT, ATF2, and NFB/Rel factors, and this collectively affects the expression of nuclear genes [77]. Mitochondrial stress can lead to uncoupling of the electron transport chain and reduced ATP levels, which can be reversed via upregulation of the transcriptional coactivator peroxisome PGC-1 and mitochondrial genes. However, PGC-1-deficient cells and mice cannot compensate for decreased ATP levels or oxidative phosphorylation levels [78]. In malignant astrocytoma cells, decreased mtDNA copy numbers correlate with increased levels of mitochondrial POLG and mitochondrial transcription factors (TFAM, and TFB1M, and TFB2M) [79]. Mitochondrial-nuclear interactions are also found in budding yeast Saccharomyces cerevisiae that show retrograde signaling associated with nutrient sensing, TOR signaling, and aging [80].

Our study uses the cybrid model to demonstrate that the expression of complement regulators (inhibitors and activators) can be mediated differentially by mitochondria from AMD subjects versus age-matched Normal subjects. At the present time, the mechanisms of this mitochondrial-nuclear signaling are not known but are under investigation. One possibility is that the mtDNA variants, e.g., (a) haplogroup defining SNPs, (b) private SNPs (non-haplogroup defining), or (c) unique SNPs (those that have not been identified in any mtDNA database) may mediate the nuclear genes. It has been shown in mitochondrial diseases (e.g., Leber hereditary optic neuropathy) that single SNP changes can greatly influence cellular functions [81,82]. In future studies, we may be able to identify SNPs that are present in AMD mtDNA but lacking in the age-matched Normal mtDNA. However, conducting these studies will be a challenge as it will require in depth sequencing of large numbers of samples, along with complex bioinformatics analyses. Plans are in place to conduct these experiments but they are not part of the present investigation.

Another possible mechanism by which mtDNA may mediate the nuclear genome would be regulation via epigenetic changes in the AMD mtDNA. Bellizzi and coworkers have shown that mtDNA variants can modulate levels of total global methylation [83], and that the mtDNA control region has unique CpG methylation patterns [84]. Using the cybrid model, we have shown that mtDNA from different haplogroups (H versus J) confers different levels of total global methylation, along with altered expression patterns for acetylation and methylation genes. In addition, different expression levels for nuclear genes (CFH, EFEMP1, VEGFA and NF*k*B2) become equivalent after treatment with a methylation inhibitor (5-aza-2’-deoxycytidine) in the H haplogroup and J haplogroup cybrids [85]. These findings suggested that the mtDNA variants can mediate methylation profiles and transcription for several important pathways associated with AMD. Further analyses are needed to clarify the mechanisms involved in the AMD versus age-matched Normal cybrids and these studies are presently underway.

In conclusion, our cybrid studies provide evidence that the complement system is one of the cellular pathways affected by the mitochondrial genome. However, further studies are needed to determine the mechanisms of retrograde signaling associated with the mtDNA-mediated dysregulation of the complement system.
